# The Association between Elevated Hematocrit and Retinal Artery Occlusion in Adult Patients

**DOI:** 10.3390/jcm11206116

**Published:** 2022-10-17

**Authors:** Wei-Yu Lai, Pei-Chin Lin, Chun-Hao Yin, Kuang-Tsu Yang, En-Jie Shih, Jin-Shuen Chen

**Affiliations:** 1 Department of Ophthalmology, Kaohsiung Veterans General Hospital, Kaohsiung 81362, Taiwan; 2 Department of Pharmacy, Kaohsiung Veterans General Hospital, Kaohsiung 81362, Taiwan; 3 School of Pharmacy, College of Pharmacy, Kaohsiung Medical University, Kaohsiung 80708, Taiwan; 4 Department of Medical Education and Research, Kaohsiung Veterans General Hospital, Kaohsiung 81362, Taiwan; 5 Institute of Health Care Management, National Sun Yat-Sen University, Kaohsiung 80424, Taiwan; 6 Division of Gastroenterology and Hepatology, Department of Internal Medicine, Kaohsiung Veterans General Hospital, Kaohsiung 81362, Taiwan; 7 Division of Family Medicine, Department of Community Medicine, Kaohsiung Municipal Min-Sheng Hospital, Kaohsiung 80251, Taiwan; 8 Department of Medicine, National Taiwan University, Taipei 10617, Taiwan; 9 Institute of Biomedical Science, National Sun Yat-Sen University, Kaohsiung 80424, Taiwan; 10 School of Medicine, National Yang Ming Chiao Tung University, Hsinchu 112304, Taiwan; 11 Division of Nephrology, Department of Internal Medicine, Kaohsiung Veterans General Hospital, Kaohsiung 81362, Taiwan; 12 Deputy Superintendent, Department of Administration, Kaohsiung Veterans General Hospital, Kaohsiung 38024, Taiwan

**Keywords:** hematocrit, retinal artery occlusion, female, hypertension, stroke

## Abstract

Retinal artery occlusion (RAO) is most commonly caused by embolism. Evidence showed that hematocrit (Hct) levels are often associated with embolic events. In this study, we aim to investigate the relationship between Hct levels and RAO. This retrospective study enrolled RAO patients between January 2011 and March 2020, who were 1:4 matched by age, gender, index date, and relevant comorbidities with the non-RAO group. Patient characteristics and laboratory data were collected. Univariate conditional logistic regression was applied by estimating crude matched odds ratios to determine the relevant factors for the occurrence of RAO. Furthermore, a narrative review of the relevant study was conducted to explore the association between Hct levels and embolism. Between January 2011 to March 2020, 82 RAO patients and 328 non-RAO patients matched with age, gender, index date, comorbidities of hypertension, diabetes mellitus, dyslipidemia, chronic kidney disease, stroke, and atrial fibrillation were enrolled after excluding ineligible individuals. Conditional logistic regression analysis showed that Hct level ≥ 40% was associated with developing RAO. A forest plot showed a trend of a non-linear dose-response association between Hct levels and ischemic vascular events in male patients. Hct levels ≥ 40% in patients older than 65 years with at least six comorbidities could be associated with RAO. We suggest that older patients who have multiple comorbidities, combined with elevated Hct levels, should be informed of the possible occurrence of RAO.

## 1. Introduction

Retinal artery occlusion (RAO) is a severe retinal embolic disease causing the sudden onset of blindness with poor prognosis [1]. The disease could be further categorized as central retinal artery occlusion (CRAO) and branch retinal artery occlusion (BRAO) according to the occlusion site. Although the incidence rate of RAO was low (6.63 per 100,000 person-years), the poor life quality caused by poor vision in RAO patients, especially with CRAO, is profound [2]. The pathogenesis of RAO is primarily the embolism lodged in the arterial lumen, thus causing retinal ischemia and edema [3]. Other causes include hemodynamic or vasospasm-related impaired blood flow [4]. The disease could be further categorized as central RAO (CRAO) and branch RAO (BRAO) according to the occlusion site. Other causes of RAO include hemodynamic or vasospasm-related impaired blood flow [4]. Characteristic fundoscopic findings showed a cherry-red spot and retinal opacification with or without emboli with a refractile (cholesterol, 80%), whitish (fibrin–platelet, 14%), or large and chalk-like lesion (calcific material, 6%) [5]. The diagnosis of RAO depends on the clinical symptoms of acute vision loss, fundoscopic examination, and fluorescein angiography with a delayed filling of the retinal artery and longer arteriovenous transit time [4]. No consensus has emerged regarding the optimal treatment of RAO. All proposed treatments are of questionable efficacy, and many have uncertain risk profiles [6]. This makes identifying risk factors of RAO important.

The risk factors of RAO, such as hypertension, diabetes mellitus (DM), chronic kidney disease (CKD), carotid atherosclerosis, stroke, and atrial fibrillation, have been well discussed in previous studies [4,7,8,9]. Increasing attention has been focused on the association of hematologic factors with RAO [7]. Elevated hematocrit (Hct) levels are associated with an increased risk of arterial thrombosis [10]. Hct is one of the major determinants of blood viscosity, and elevation in the Hct might increase clot formation [11]. For example, in patients with polycythemia vera, a chronic clonal disorder associated with the excessive proliferation of erythrocytes, leukocytes, thrombocytes and characterized by elevated Hct levels, have been reported to present ocular manifestation of CRAO [12,13]. However, there have only been case reports associating Hct with RAO, and their relationship is still unclear [12,13].

We hypothesized that a common hematological test, Hct, combined with multiple underlying medical conditions, may be considered as risk factors for the development of RAO. In this study, we aimed to investigate the relationship between Hct and RAO, as well as other hematological biomarkers. We also wanted to identify the cutoff level of Hct when it is associated with RAO. Furthermore, a narrative review of relevant studies was conducted to explore the association between Hct levels and embolism.

## 2. Materials and Methods

### 2.1. Case Enrollment

We identified patients with first-occurrence of RAO by a chart review for the period from January 2011 to March 2020 from the database of Kaohsiung Veterans General Hospital. RAO was confirmed by an ophthalmologist based on the classic findings as follows: (i) a history of a sudden onset of visual deterioration in the eye and (ii) evidence of acute retinal ischemia in the distribution of the occluded retinal artery on the initial ophthalmic evaluation. We excluded those without lab data availability within 6 months of the index date and 1 month after the index date. The RAO study group was then matched by age, sex, index date, and comorbidities, namely, hypertension, DM, dyslipidemia, stroke, CKD, and atrial fibrillation in a 1:4 ratio with the non-RAO group. The index date was the date on which the patients were first diagnosed with RAO. For the non-RAO group, the index date was simply a matched date in which controls had a medical utilization. This human subject research was approved by the Institutional Review Board (KSVGH110-D01-1) of Kaohsiung Veterans General Hospital in Taiwan. Our study is a retrospective study, and it meets the criteria for a waiver of informed consent in accordance with the institutional requirements: the waiver will not adversely affect the rights of the subjects, and the research involves no more than minimal risk to the patients.

### 2.2. Data Collection

We recorded data for comorbidities, namely, hypertension, DM, dyslipidemia, stroke, CKD, and atrial fibrillation, because these conditions are critical risk factors for RAO [4]. In this study, the inclusion criterion for the comorbidities was the documentation of the condition at least once in an inpatient or outpatient setting. The available laboratory data were collected, including white blood cell, hemoglobin (Hb), Hct, platelet count, estimated glomerular filtration rate (eGFR), blood urea nitrogen, glutamic-pyruvic transaminase, triglyceride, high-density lipoprotein cholesterol, low-density lipoprotein cholesterol, and hemoglobin A1c, which were obtained within 6 months of the index date and 1 month after the index date.

### 2.3. Statistical Analysis

We performed all statistical analyses using SPSS statistical software, version 20 (SPSS Inc., Chicago, IL, USA) and SAS (version 9.4; SAS System for Windows, SAS Institute, Cary, NC, USA). Patient characteristics, comorbidities, and laboratory tests were compared between the RAO and non-RAO groups. Categorical variables were analyzed using the Pearson chi-square or Fisher exact test, and continuous variables were compared using one-way analysis of variance. A matched case-control study was conducted through a propensity score match to balance the baseline characteristics between groups. Propensity scores provide a method for matching on multiple confounding variables, without the limitations of the covariate matching method [14]. The propensity score match was performed at a ratio of 1:4, matching by age, sex, index year, and relevant comorbidities to minimize the effect of confounding factors. The matched data was regressed with conditional logistic regression. The univariate conditional logistic regression was applied by estimating crude matched odds ratios and their 95% confidence intervals to compare cases and controls for different factors. A *p*-value < 0.05 was considered statistically significant.

### 2.4. Narrative Reviews on the Association between Hct Levels and Embolism

We performed a literature search using the online database about the association between Hct levels and embolism for the period of 1 January 2000 through 1 November 2020. According to previous literature, both thrombi and emboli could cause myocardial infarction (MI) [15,16] and ischemic stroke [17,18]. The relationship between RAO and stroke [4,19] or MI [4,20,21] has been suggested. Thus, we may use the association of Hct and MI as well as ischemic stroke to assume the correlation between Hct and RAO, the severe retinal embolic disease. We conducted the literature search using the following keywords and MeSH terms: “hematocrit”, “emboli”, “thromboembolism”, “ischemic stroke”, “cerebrovascular”, and “cardiovascular”. In addition, the bibliographies of retrieved studies and recent review articles were checked for additional relevant studies. We included clinical or observational studies and extracted the data that represents the association between Hct levels and embolism or mortality outcomes. The forest plots were used to systematically represent the association between Hct levels and embolism.

## 3. Results

### 3.1. Comparison of RAO and Non-RAO Patients in Terms of Demographic Data and Available Laboratory Data

**Figure 1:**
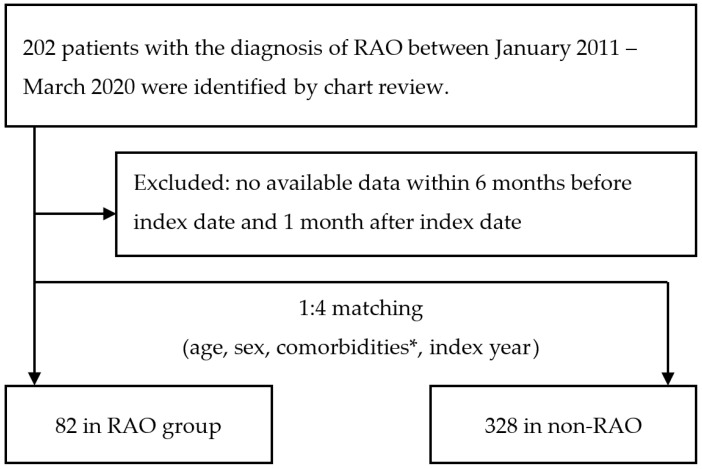
Flow chart of the patient selection process. RAO, retinal artery occlusion; ICD, international classification of diseases. * Note: the comorbidities included hypertension, diabetes mellitus, dyslipidemia, chronic kidney disease, stroke, and atrial fibrillation.

Between January 2011 to March 2020, 82 RAO patients and 328 age-, sex-, index-date-, and comorbidities-matched non-RAO patients were enrolled after excluding ineligible individuals. The patient flow is displayed in Figure 1. Table 1a shows the characteristics of the study cohorts. We tried to obtain a matched case-control study through a propensity score with the most matched baseline characteristics. Therefore, in the process, age was unable to be matched. RAO patients were younger than non-RAO patients (65.1 ± 16.6 vs. 69.8 ± 15.1, *p* = 0.013). Sex and comorbidities were similar between the two groups (*p* > 0.05 for all). RAO patients had a higher Hb level (13.4 ± 1.9 vs. 12.8 ± 2.0, *p* = 0.015), and Hct level (39.9 ± 5.3 vs. 38.2 ± 5.8, *p* = 0.021) compared with non-RAO patients. Because of missing data on high-density lipoprotein cholesterol, low-density lipoprotein cholesterol, hemoglobin A1c, and triglyceride in the study cohorts, these results were not presented. Table 1b shows the fundus change of RAO patients. Retinal opacification in the posterior pole is predominant (95%), followed by a cherry-red spot (62.5%) and arterial attenuation (50%). Disc edema upon examination is also common (37.5%). Observation of emboli is infrequent (7.5%).

### 3.2. Factors Associated with RAO

The results of the conditional logistic regression analyses of associating factors for RAO are presented in Table 2. There was no significance between the categorized age groups in RAO. Hct level ≥ 40% (odds ratio, 1.74; 95% confidence interval, 1.01–3.02, *p* < 0.046) was the only significant hematologic parameter associated with the occurrence of RAO.

### 3.3. Narrative Review of the Association between Hct and Embolism

**Figure 2:**
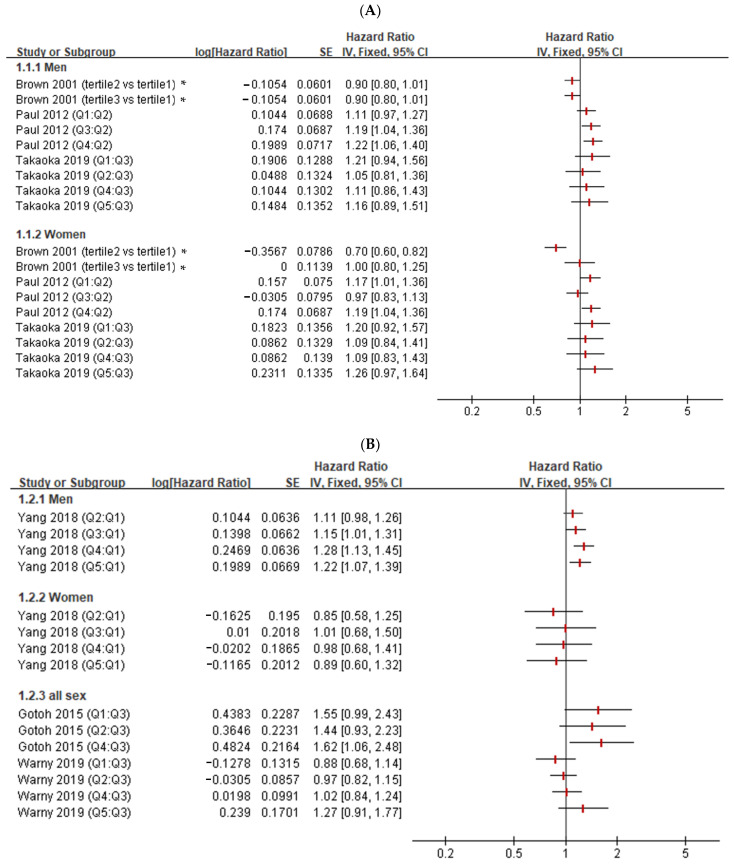

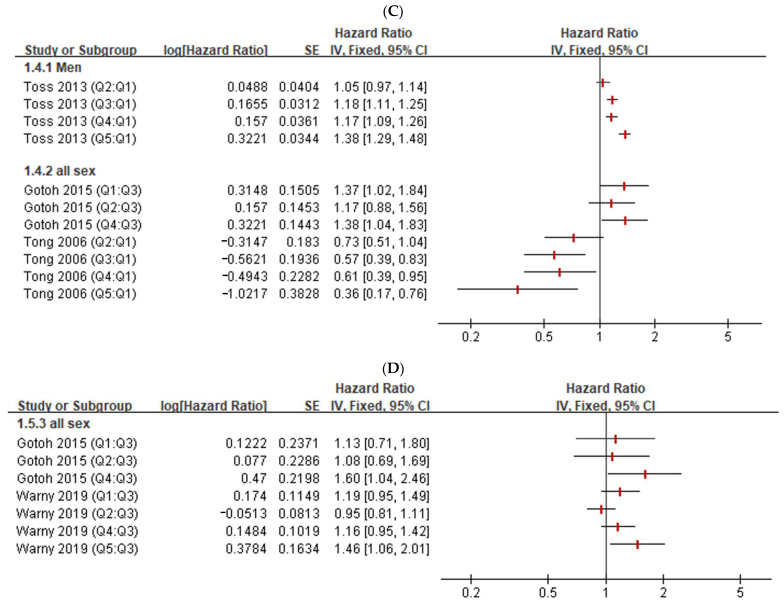
The association between hematocrit levels and diseases related to embolism. (**A**) Mortality (all cause, acute myocardial infarction, or acute stroke-related) [22,24,28]; (**B**) ischemic stroke [26,27,29]; (**C**) cardiovascular diseases (include arterial embolism in the heart or brain) [23,25,26]; (**D**) coronary heart disease [26,29]. SE = standard error; IV = inverse variance; CI, confidence interval * Note: the effect size for Brown’s (2001) study was a relative risk rather than the hazard ratio in other studies.

The initial literature search identified 34 articles through a systematic search in PubMed in November 2020. We further excluded 27 studies because they were in vitro or in vivo studies with different aims, reviews, or case reports. The remaining seven studies were subjected to a complete full-text review. Moreover, one study was retrieved from hand-searching. Finally, a total of eight studies were included [22,23,24,25,26,27,28,29]. Even when the definition of quartiles was heterogeneous, all studies, except those by Brown et al., Paul et al., and Gotoh et al. [22,24,26], categorized the Hct levels into five quintiles, with 1 (Q1) being the lowest and 5 (Q5) being the highest. There was a non-linear dose-response association between Hct levels and mortality or ischemic vascular events in males (Figure 2A–C), but no definite association was found in females. However, only one study presented the association between Hct levels and hemorrhagic stroke (Figure 2C). Ischemic stroke, hemorrhagic stroke, cardiovascular diseases (include arterial embolism in heart or brain), and coronary heart disease were not found to be related to Hct levels in studies including participants of both sexes (Figure 2B–D). In summary, a non-linear dose-response association between Hct levels and mortality or ischemic vascular events were found in males but not in females.

## 4. Discussion

In this study, we controlled our analyses for age, sex, and comorbidities, namely, hypertension, DM, dyslipidemia, stroke, CKD, and atrial fibrillation; however, the association between Hct levels and RAO was independent of these potential confounders. Hct level ≥ 40% is 1.74 times more likely to be associated with the occurrence of RAO regardless of age, sex, and the above comorbidities. Additionally, we reviewed the published data, which showed there is a non-linear dose-response association between Hct levels and mortality or ischemic vascular events in males, but no definite association in females was found [22,23,24,25,26,27,28].

Current reports showed that an extremely high Hct level may be associated with RAO [12,13,30,31,32,33,34]. Feng et al., has reported a case of BRAO in a high-altitude male climber with a Hct level of 52.4%; in addition, the man also had DM, which might be a predisposing factor for RAO [30]. Polycythemia vera, a hyperviscosity disease reflected in increased Hct, have been reported to cause RAO [12,13,31,32]. In these cases, the Hct levels were more than 50%. Another hematologic disorder, namely, essential thrombocytopenia has been associated with RAO [32]. A young man who developed RAO was later diagnosed with essential thrombocytopenia. His platelet count level was >1,000,000 mm^3^, with a Hct level of 48.3% [33]. In our study, when the comorbidities were controlled, Hct > 40% was associated with RAO.

The association between higher level of Hct with systemic thromboembolic events have also been reported in previous literature. Brakkan et al., found that men with Hct levels ≥ 46% had a 1.5-fold increased risk of total venous thromboembolism compared to men with Hct levels < 39% [10]. However, because of a lack of available information, they did not consider underlying medical conditions. In the Chinese city Kailun, Yang et al., found that males with Hct levels > 43.2% had a 1.7-fold higher risk of ischemic stroke than males with Hct levels < 41.5%, after adjusting for age, sex, smoking, drinking, body mass index, hypertension, DM, dyslipidemia, atrial fibrillation, systolic blood pressure, fasting plasma glucose, total cholesterol, low-density lipoprotein, eGFR, and proteinuria [27]. In a Japanese population, after adjusting for age, sex, diastolic blood pressure, DM, total cholesterol, body mass index, albumin, electrocardiogram abnormalities, eGFR, smoking habits, alcohol intake, and regular exercise, the highest (males ≥ 43.8%, females ≥ 43.8%) and lowest (males ≤ 44.7%, females ≤ 39.3%) Hct quartiles were significantly associated with the development of ischemic stroke [26]. 

Multiple comorbidities have a well-known association with retinal emboli [5,35,36]. Common cardiovascular risk factors, including sex, age, arterial hypertension, DM, dyslipidemia, atrial fibrillation, CKD, and stroke, have been described as more significantly found among RAO patients than in the general population [37]. These diseases may cause microcirculation disorder and indirectly increase the risk of RAO. In reality, more people are living longer with the concurrence of two or more chronic medical conditions [38]. Therefore, we believe it is important to compare the baseline Hct levels between patients with similar comorbidities. 

The strength of our study includes the ability to control several important medical conditions. However, this study had a few limitations. First, due to the retrospective design in our study, we excluded several patients due to non-available laboratory data and several common laboratory data for atherosclerosis (triglyceride, high-density lipoprotein cholesterol, low-density lipoprotein cholesterol, and hemoglobin A1c). Further studies including a larger number of patients with hematological data are necessary to investigate the role of hematological biomarkers in RAO disease. Second, our finding was based on the mean measurement of Hct levels checked within 6 months of the index date or 1 month after the index date. Therefore, we could not account for variations in Hct levels due to hydration status and their potential association between RAO.

In conclusion, we found that Hct level ≥ 40% is associated with the occurrence of RAO in patients with at least six comorbidities. We suggest that patients older than 65 years with Hct levels > 40% and multiple comorbidities should be made aware of the possible development of RAO. In addition, our narrative review presented with a forest plot provides current published data on the association between Hct levels and emboli.

## Figures and Tables

**Table 1:** **a.** Baseline characteristics of RAO patients, *n* = 410. **b**. Fundus change of RAO patients.

	Total (*n* = 410)	RAO (*n* = 82)	Non-RAO (*n* = 328)	*p* Value
Age, years (mean ± SD)	68.9 ± 15.5	65.1 ± 16.6	69.8 ± 15.1	0.013
Gender (%)				0.497
Male	272 (66)	57 (70)	215 (66)	
Female	138 (34)	25 (31)	113 (35)	
Comorbidities (%)				
Hypertension	247 (60)	47 (57)	200 (61)	0.545
Diabetes mellitus	101 (25)	23 (28)	78 (24)	0.422
Dyslipidemia	154 (38)	32 (39)	122 (37)	0.760
Chronic kidney disease	31 (8)	4 (5)	27 (8)	0.304
Stroke	151 (37)	27 (33)	124 (38)	0.413
Atrial fibrillation	41 (10)	8 (10)	33 (10)	0.934
Hematology parameters *				
WBC (1000/µL)	8.0 ± 6.7	7.5 ± 2.2	8.2 ± 7.5	0.384
Hb (g/dL)	12.9 ± 2.0	13.4 ± 1.9	12.8 ± 2.0	0.015
Hct (%)	38.6 ± 5.7	39.9 ± 5.3	38.2 ± 5.8	0.021
Platelet (1000/µL)	222.6 ± 71.6	223.4 ± 64.6	222.5 ± 73.4	0.911
eGFR (ml/min/1.73 m^2^)	70.7 ± 24.1	69.0 ± 25.2	71.2 ± 23.8	0.480
GPT (U/L)	32.2 ± 48.1	25.2 ± 19.4	34.0 ± 52.8	0.136
**Fundus Change (%)**	**CRAO/BRAO (*n* =** **82** **)**			
Retinal opacification	78 (95)			
Cherry-red spot	58 (71)			
Artery attenuation	40 (49)			
Disc edema	15 (18)			
Box carring	15 (18)			
Emboli	6 (7)			

RAO, retinal artery occlusion; WBC: white blood cell; Hb: Hemoglobin; Hct: hematocrit; eGFR: estimated glomerular filtration rate; GPT: glutamic pyruvic transaminase. * Note: the hematology parameters were measured within 6 months before RAO diagnosis and 1 month after RAO diagnosis. CRAO, central retinal artery occlusion; BRAO, branch retinal artery occlusion.

**Table 2:** Conditional logistic regression analysis for RAO patients.

Variables	Beta	OR (95% CI)	*p* Value
Age			
<40	0.889	2.44 (0.79–7.48)	0.120
40–55	0.506	1.66 (0.77–3.60)	0.200
55–65	0.214	1.24 (0.60–2.56)	0.563
>65	1	1	1
Hematology parameters			
WBC < 9 (1000/µL)	0.161	1.17 (0.64–2.15)	0.603
eGFR ≤ 60 (ml/min/1.73 m^2^)	0.400	1.49 (0.84–2.64)	0.170
Platelet <215 (1000/µL)	0.158	1.17 (0.71–1.94)	0.539
GPT ≤ 30 (U/L)	0.507	1.66 (0.94–2.92)	0.079
Hct ≥ 40 (%)	0.556	1.74 (1.01–3.02)	0.046
Hb ≥ 13 (g/dL)	0.389	1.48 (0.84–2.58)	0.172

RAO, retinal artery occlusion; OR, odds ratio; CI, confidence interval; WBC: white blood cell; Hb: Hemoglobin; Hct: hematocrit; eGFR: estimated glomerular filtration rate; GPT: glutamic pyruvic transaminase. Note: the hematology parameters were measured within 6 months before RAO diagnosis and 1 month after RAO diagnosis.

## Data Availability

All data are available upon a reasonable request from the authors.
